# Dynamics of Bacterial Communities Mediating the Treatment of an As-Rich Acid Mine Drainage in a Field Pilot

**DOI:** 10.3389/fmicb.2018.03169

**Published:** 2018-12-21

**Authors:** Elia Laroche, Corinne Casiot, Lidia Fernandez-Rojo, Angélique Desoeuvre, Vincent Tardy, Odile Bruneel, Fabienne Battaglia-Brunet, Catherine Joulian, Marina Héry

**Affiliations:** ^1^HydroSciences Montpellier, CNRS, IRD, University of Montpellier, Montpellier, France; ^2^BRGM, Geomicrobiology and Environmental Monitoring Unit, Orléans, France

**Keywords:** acid mine drainage, arsenic, bioremediation, eco-engineering, iron-oxidizing bacteria, arsenic-oxidizing bacteria, microbial ecotoxicology

## Abstract

Passive treatment based on iron biological oxidation is a promising strategy for Arsenic (As)-rich acid mine drainage (AMD) remediation. In the present study, we characterized by 16S rRNA metabarcoding the bacterial diversity in a field-pilot bioreactor treating extremely As-rich AMD *in situ*, over a 6 months monitoring period. Inside the bioreactor, the bacterial communities responsible for iron and arsenic removal formed a biofilm (“biogenic precipitate”) whose composition varied in time and space. These communities evolved from a structure at first similar to the one of the feed water used as an inoculum to a structure quite similar to the natural biofilm developing *in situ* in the AMD. Over the monitoring period, iron-oxidizing bacteria always largely dominated the biogenic precipitate, with distinct populations (*Gallionella, Ferrovum, Leptospirillum, Acidithiobacillus, Ferritrophicum*), whose relative proportions extensively varied among time and space. A spatial structuring was observed inside the trays (arranged in series) composing the bioreactor. This spatial dynamic could be linked to the variation of the physico-chemistry of the AMD water between the raw water entering and the treated water exiting the pilot. According to redundancy analysis (RDA), the following parameters exerted a control on the bacterial communities potentially involved in the water treatment process: dissolved oxygen, temperature, pH, dissolved sulfates, arsenic and Fe(II) concentrations and redox potential. Appreciable arsenite oxidation occurring in the bioreactor could be linked to the stable presence of two distinct monophylogenetic groups of *Thiomonas* related bacteria. The ubiquity and the physiological diversity of the bacteria identified, as well as the presence of bacteria of biotechnological relevance, suggested that this treatment system could be applied to the treatment of other AMD.

## Introduction

Mining activities produced large amount of wastes composed of sulfide minerals and toxic metallic elements, causing environmental hazard. During their inappropriate storage, oxidation of the sulfide minerals can generate acid mine drainage (AMD) characterized by acid pH and high concentrations in metals and metalloids. Arsenic (As) is a highly toxic metalloid often associated with this type of pollution (Paikaray, 2015). An AMD can self-sustain for several centuries, with dramatic consequences on the receiving aquatic ecosystems. In this context, the development of effective and sustainable remediation solution for the treatment of As-rich AMD is required for water resource and public health protection.

The dissemination of arsenic pollution can be naturally limited by its immobilization into the solid phase. This natural phenomenon relies on the biological oxidation of Fe(II) and its subsequent precipitation as Fe(III) oxyhydroxides. Arsenic is then removed from the dissolved phase by co-precipitation with Fe(III) or by adsorption to the newly formed ferric minerals. The less soluble form, arsenate [As(V)], is more efficiently trapped onto iron phases than the more mobile form, arsenite [As(III)] (Hug and Leupin, 2003). Then, the capacity of autochthonous bacteria to oxidize As(III) into As(V) together with the activity of iron-oxidizing bacteria (FeOB) contribute to the mitigation of the arsenic pollution. This natural attenuation has been described at various mining sites worldwide (Casiot et al., 2003; Asta et al., 2010; Egal et al., 2010; Chen and Jiang, 2012; Paikaray, 2015), as well as the diversity of AMD autochthonous microbial communities (Baker and Banfield, 2003; Bruneel et al., 2011; Volant et al., 2014; Méndez-García et al., 2015; Chen et al., 2016). Several studies have focused on the identity of FeOB and As(III)-oxidizing bacteria (AsOB) and their activity in relation with pollution attenuation (Battaglia-Brunet et al., 2002; Bruneel et al., 2003; Casiot et al., 2003; Duquesne et al., 2003; Egal et al., 2009). In particular, numerous evidences of the role of the As(III)-oxidizing *Thiomonas* spp. were reported (Bruneel et al., 2003, 2011; Casiot et al., 2003; Hovasse et al., 2016).

The development of passive treatment systems exploiting these microbially mediated processes is a promising strategy for the remediation of As-rich AMD (Hengen et al., 2014; Bruneel et al., 2017). The efficiency of such system was demonstrated in lab-scale bioreactors (González-Contreras et al., 2012; Hedrich and Johnson, 2014; Ahoranta et al., 2016; Fernandez-Rojo et al., 2017). Attempts of *in situ* treatment are scarce and mainly limited so far to AMD with arsenic concentrations lower than 3 mg L^-1^ (Whitehead et al., 2005; Macías et al., 2012). One field-pilot treating high arsenic concentrations (50–250 mg L^-1^) removed 20% of the dissolved arsenic (Elbaz-Poulichet et al., 2006). The treatment efficiency of these systems relies on microbial activity and thus may fluctuate depending on the identity and the dynamic of the microbial populations driving the biogeochemical reactions inside the bioreactor. Operating conditions as well as environmental conditions likely exert control over microbial diversity and activity (Heinzel et al., 2009b; Ahoranta et al., 2016; Fernandez-Rojo et al., 2017).

In a previous study, a passive field-scale bioreactor treating As-rich acid mine drainage from the Carnoulès mine (France) was monitored during 6 months (Fernandez-Rojo et al., in press). Arsenic removal varied between 3 and 97% during the monitoring period for a flow rate variation between 6 and 130 L h^-1^. The proportion of As(V) in the biogenic precipitates formed inside the pilot increased over time, reaching nearly 100% of total As. Arsenate enrichment in the precipitate was associated with an increase of the abundance of the marker gene for bacterial arsenite oxidation (*aioA*) -quantified by qPCR-. Results strongly suggested that bacterial As(III) oxidation occurred in the bioreactor, leading to the formation of stable precipitates, advantageous in term of sludge management. However, the identity of the bacterial populations involved in arsenic and iron oxidation and removal were not determined. The aim of the present work was to improve our knowledge of the bacterial diversity driving As-rich AMD depollution in an *in situ* treatment device. In this context, the spatial and temporal dynamics of the bacterial communities colonizing the bioreactor were investigated by a 16S rRNA metabarcoding approach. Because of their central role in arsenic oxidation in Carnoulès AMD, we further characterized two distinct monophyletic *Thiomonas* groups by CARD-FISH. We also focused on the physico-chemical drivers of the bacterial diversity. To our knowledge, this is the first microbiological characterization performed on a field-pilot treating As-rich AMD by precipitation with biogenic iron phases.

## Materials and Methods

### Pilot Scale Passive Treatment System Description and Sample Collection

**FIGURE 1:**
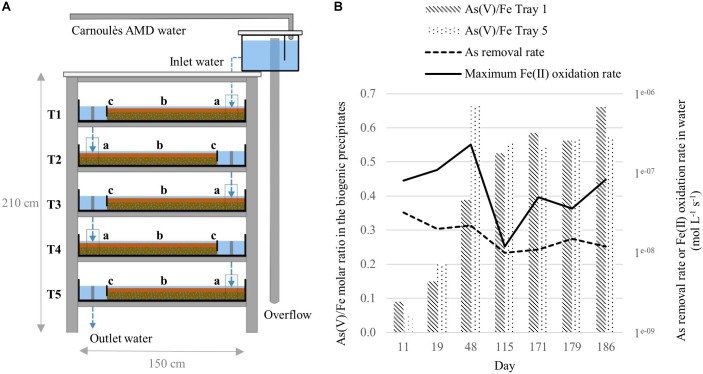
Schematic representation of the field-bioreactor composed of five trays (T = tray) adapted from Fernandez-Rojo et al. (in press) **(A)**. Arsenic removal rates calculated from inlet and outlet dissolved As concentration and estimated hydraulic retention time and maximum Fe(II) oxidation rates estimated from analytical uncertainty on dissolved Fe(II) determination, according to Fernandez-Rojo et al. (in press) **(B).**

A field-scale pilot, made with five shallow trays (1.5 × 1 × 0.11 m) stacked head to tail on a shelf, was implemented at the Carnoulès mine (France) for the treatment of AMD, as described in Fernandez-Rojo et al. (in press) (Figure 1A). The bioreactor was settled on June 2016 10th and was monitored during 194 days. Nine sampling campaigns were conducted and inlet water and biogenic precipitates were characterized for their physico-chemical properties (Fernandez-Rojo et al., in press). From the whole dataset and samples available, we selected seven dates for microbial characterization: 21st of June (D11); 29th of June (D19); 28th of July (D48); 3rd of October (D115); 28th of November (D171), 6th of December (D179), 13th of December (D186). Selection was based on the temporal variations of the As(V)/Fe molar ratio in the biogenic precipitate (Figure 1B). Arsenic removal rates from water and Fe(II) oxidation rates are also given. Biogenic precipitates were collected from tray 1 (T1) and tray 5 (T5) for all the campaigns and from tray 1, 2, 3, 4, and 5 for the D48 and D171 campaigns.

Each tray was divided into three sections (Figure 1A): “a” refers to the location close to the inlet of the tray, “b” corresponds to the middle section, and “c” refers to the location close to the outlet of the tray. Based on ARISA (Automated Ribosomal Intergenic Spacer Analysis), bacterial community structures in the three sections (a, b, c) of a given tray exhibited similar genetic structure (data not shown). Then, for each tray, samples collected in sections a, b and c were used as triplicates for 16S rRNA metabarcoding analyses. For each tray, three composite samples were collected in Falcon tubes (50 mL) by scrapping the biogenic precipitates in each section with a sterile spatula. The tubes containing the composite samples were centrifuged for 10 min at 4400 × *g* (Sorwall ST40, Thermo Scientific). The supernatant was discarded and the precipitates were homogenized. An aliquot was dried in a vacuum desiccator for dry weight determination. The remaining precipitates were conserved in Eppendorf tubes (2 mL) at -80°C before DNA extraction. Sample names refer to the sampling day (counted from the day of bioreactor settling, D1–D186) and the tray where the biogenic precipitate was sampled (T1–T5), e.g., D11-T1.

Inlet water was collected for all the campaigns but due to technical problem, water collected on October 3rd (D115) could not be analyzed. Triplicates of inlet water samples (300 mL) were filtered on sterile 0.22 μm cellulose acetate filter. The filters were stored at -80°C before DNA extraction. Water sample names refer to the sampling day (D1–D186) of inlet water sample (W_in_) (e.g., D11-W_in_).

For comparison between the bacterial communities established inside the bioreactor and the natural ecosystem, a point-in-time sampling of the AMD riverbed sediments (Reigous creek) was performed on the 28th of November (D171). Sediments (SED) were collected using a sterile spatula and were treated as described for the biogenic precipitates.

### Physico-Chemical Characterization of Inlet and Outlet Waters

The chemical characterization of the water (inlet and outlet) and the bioprecipitate samples was performed in our previous study (Fernandez-Rojo et al., in press). Briefly, the concentrations of dissolved Fe(II) were determined using spectrophotometry after filtration through 0.22 μm. Total dissolved concentrations of Fe, As, and other elements [S (SO_4_^2-^), Al, Zn, and Pb] were determined using ICP-MS. Arsenic redox speciation was determined using HPLC-ICP-MS. The data used in the present study are summarized in Figure 1B and Supplementary Table S1.

### DNA Extraction and Quantification

Prior DNA extraction, biogenic precipitate and riverbed sediment samples were washed with 1 mL of TRIS-EDTA (100:40) HCl pH 8 and then centrifuged to 8,000 × *g* from 1 min. Total genomic DNA was then extracted using the DNeasy PowerSoil kit (Qiagen, Hilden, Germany), according to the manufacturer’s recommendations (in triplicates for the biogenic precipitates). Extraction controls were performed without any biogenic precipitate sample to exclude any contamination from the kit. For water samples, DNA was extracted (in triplicates) from the cellulose acetate filters using DNeasy PowerWater kit (Qiagen) according to the manufacturer’s recommendations. All the DNA extracts were quantified with a fluorometer (Qubit, Invitrogen, Carlsbad, CA, United States) and stored at -20°C until further analysis.

### Sequencing of Bacterial 16S rRNA Gene

The V4–V5 hypervariable region of the 16S rRNA gene was amplified using primers PCR 515F (Barret et al., 2015) and PCR 928R (Wang and Qian, 2009). PCR amplification was conducted as described in Tardy et al. (2018). Amplification performed on the control DNA extracts (obtained without any biogenic precipitate sample) yielded no amplification signal. The PCR products were sequenced with an Illumina MiSeq sequencer in paired-end mode (2 × 300 bp) at GeT-PlaGe platform (Toulouse, France).

### Bioinformatic Analyses of 16S rRNA Gene Sequences

Pair-end sequences were merged by flash software version 1.2.6 (Magoè and Salzberg, 2011), with maximum 10% of mismatch into the overlap region. The raw datasets are available on the EBI database system under project accession number [PRJEB27907]. The average Phred score (*Q*-score) of these joined reads was superior to 30 for every base. Bioinformatic analyses were conducted using the software program Mothur version 1.39.5 (Schloss et al., 2009). First step was the selection of high quality sequences based on the following criteria: length between 330 and 460 bp, a homopolymer length inferior to 7 nt and no ambiguous bases. Singletons, chimeric and unaligned reads were removed using UCHIME (Edgar et al., 2011) and the SILVA reference database (Release 128). The pre-cluster command served to reduce sequencing noise by clustering reads differing by only one base every 100 bases. The high quality sequences were taxonomically assigned using the SILVA reference database, by the Bayesian method with a bootstrap confidence score of 80 (Wang et al., 2007). After the removal of sequences that were not assigned to the *Bacteria* kingdom, samples contained between 2227 and 34928 reads. A subsampling selected a random set of 6295 reads in each sample in order to efficiently compare the datasets. Samples containing less than 6295 reads were removed to maintain a sequencing effort that adequately covers the bacterial diversity. A distance matrix was generated with the remaining sequences and used to cluster these sequences into Operational Taxonomic Units (OTUs) defined at 97% cutoff, using the average neighbor algorithm. Complementary analyses (based on BLAST) were performed for the assignation of the dominant OTU (representing 41% of the whole reads) at the genus level. Mothur used the OTU table to calculate the coverage sample (rarefaction curve), the alpha and beta diversity (diversity indices and Unifrac distances) at a level of 97% sequence similarity.

### CARD – FISH

CARD-FISH analyses were performed in triplicates on biogenic precipitates collected from tray 1 (T1) and tray 5 (T5) on the 28^th^ of July (D48) and the 28th of November 2016 (D171). Oligonucleotide probes EUB338, EUB338II and EUB338III were used for *Bacteria* quantification (Amann et al., 1990; Daims et al., 1999). Negative controls were performed with probe NON338, the complementary sequence of EUB338 (Wallner et al., 1993). Two probes were used for the quantification of two distinct monophyletic groups of *Thiomonas* spp. Probes TM1G0138 and TM2G0138 respectively target *Thiomonas* group1 and group2 according to Hallberg et al. (2006), which correspond respectively to monophyletic Group II and I according to Coupland et al. (2004) and Bryan et al. (2009). Detailed protocol for *in situ* hybridization, tyramide amplification, and microscopic observations is provided in Supplementary File.

### Statistical Analyses

All statistical analyses were performed with R version 3.4.3 using mainly vegan package^1^. The statistically significant differences between CARD-FISH data were assessed with the non-parametric Kruskal–Wallis test followed by Dunn’s *post hoc* test using Bonferroni *p*-value adjustment. Non-metric multidimensional scaling (NMDS), based on the weighted pairwise Unifrac distances, was used to illustrate the dynamics of the bacterial communities structure during the monitoring. The significance of the differences observed between groups of samples (phase I vs. phase II and tray 1 vs. tray 5) was assessed with an ADONIS test (999 permutations). Redundancy analysis (RDA) was performed to highlight the main environmental drivers shaping the bacterial community structure inside the bioreactor. This approach was chosen because an initial detrended correspondence analysis (DCA) indicated that bacterial community data had a linear distribution along the axis (<3.0). The physico-chemical characteristics of the AMD water, measured in the inlet and outlet waters (Supplementary Table S1), were tested as environmental variables to explain the variations of dominant taxa in the bioprecipitates collected in tray 1 and tray 5 respectively (relative abundance of reads ≥ 1% in at least one bioprecipitate sample during the monitoring period). Indeed, the biofilm continually interacts with the inflowing water which provides the main energy source for the lithotrophic bacteria [Fe(II)]. Before the RDA, the normality of environmental variables was checked with a Shapiro–Wilk test. Environmental and biological variables were respectively log and Hellinger transformed (Legendre and Gallagher, 2001). A sub-set of environmental variables was selected to decrease the multicolinearity (variance inflation factors < 10) and to include only the significant predictors (*p* < 0.05 according Monte Carlo test with 999 permutations). Variation partitioning analyses enabled to evaluate the contribution of each variable to the bacterial community variation. The significance of the global model and the individual axes were checked with a Monte Carlo test (999 permutations).

## Results

### Bacterial Diversity and Structure in the Treatment Pilot

Illumina sequencing of the 79 samples yielded a total of 2,288,823 sequences of 16S rRNA gene. Among them, 6295 quality sequences per sample were sub-sampled for alpha and beta diversity analysis. Rarefaction curves approached an asymptote suggesting that the sequencing effort adequately covered the bacterial diversity in all the samples (Supplementary Figure S1).

**FIGURE 2:**
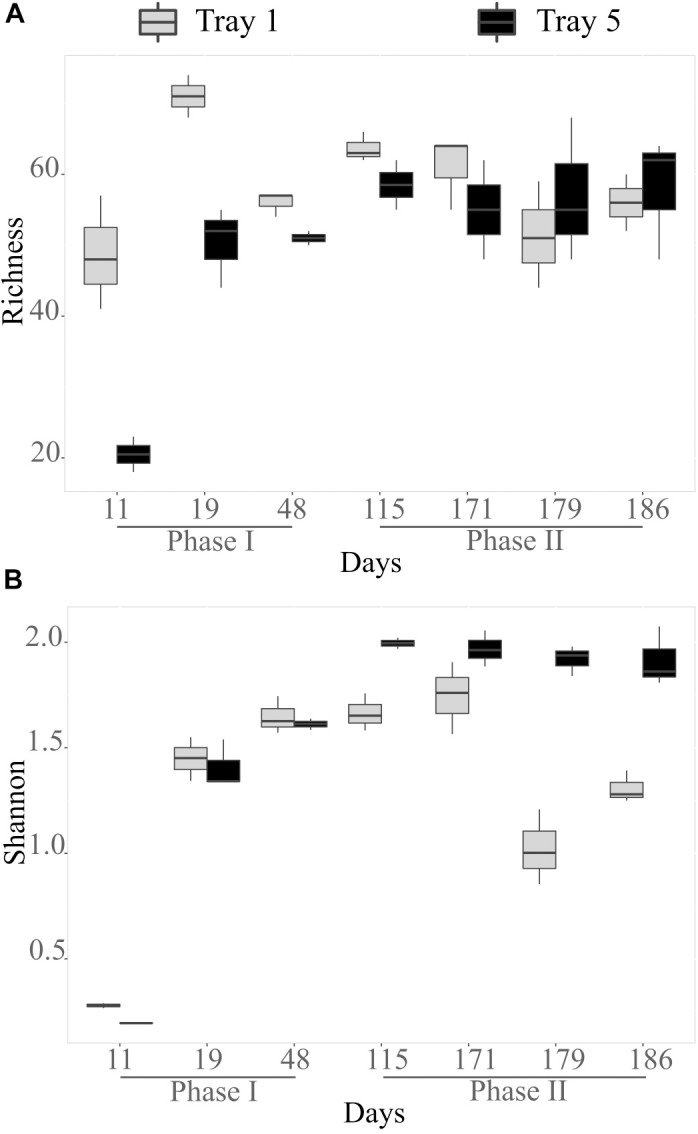
Bacterial species richness in number of OTUs observed **(A)** and Shannon diversity index **(B)** in the biogenic precipitates collected in the tray 1 (light gray) and tray 5 (black). Error bars represent standard deviation of the mean value (analyses performed in triplicates).

In the biogenic precipitates, the richness increased from D11 to D19 as illustrated by the enrichment of observed OTUs (Figure 2A). At D11 and D19, richness was higher in the top tray than in the bottom tray. From D48, the number of observed OTUs remained relatively stable and similar in both trays. Shannon index (reflecting both richness and evenness) also clearly increased during the first stages of the monitoring. Then it remained stable in tray 5 from D115 to D186, whereas it tended to decrease before increasing again in tray 1 (Figure 2B). Diversity indexes in the sediments collected in the AMD riverbed on day 171 (point in time analysis) were comparable to those measured in the biogenic precipitates collected at the same date in the top tray (T1) (richness = 60 observed OTUs; Shannon = 1.87).

**FIGURE 3:**
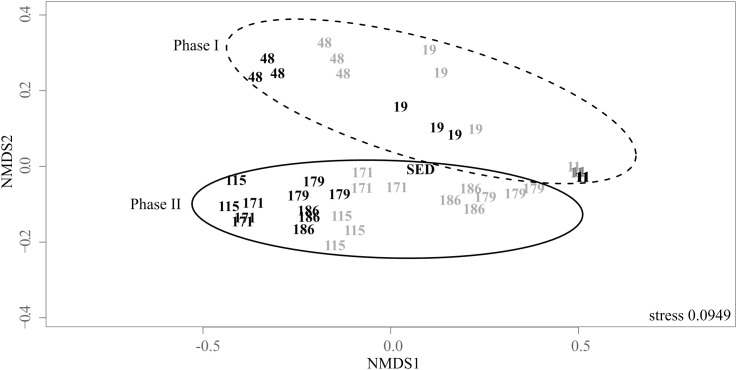
Non-metric multidimensional scaling (NMDS) ordination plot of the weighted pairwise Unifrac distances of the bacterial community in the biogenic precipitates collected on the seven campaigns (the numbers indicate the days of sampling) in the tray 1 (gray) and the tray 5 (black) of the bioreactor (analyses performed in triplicates) and in the riverbed sediment (SED) collected on day 171. Stress values for ordination plot were < 0.2 which indicates that these data were well-represented by the two-dimensional representation.

NMDS illustrates the dynamics of the structure of the bacterial communities in the biogenic precipitates and in the riverbed sediment (SED) (Figure 3). Samples were separated in two main groups (ADONIS test, *R*^2^ = 0.19, *P* = 0.001). The first group, composed by the communities at D11, D19, and D48, corresponded to the first phase of the monitoring (phase I). The second group, composed by the communities at D115–D186 corresponded to the second phase of the monitoring (phase II). In addition, over the whole monitoring period, the bacterial communities in the top tray (tray 1) were distinct from those thriving in the bottom tray (tray 5) (ADONIS test, *R*^2^= 0.14, *P* = 0.012). This spatial structuring, visible from D48, became particularly marked from D115. Bacterial community thriving in the sediment collected in the AMD riverbed (SED) grouped with those of the biogenic precipitates collected at the same date (D171) in the top tray (Figure 3).

### Taxonomic Composition of the Bacterial Communities

The majority of the 16S rRNA gene sequences retrieved from the biogenic precipitates, the riverbed sediments, and the AMD waters were affiliated with members of the *Beta*-*Proteobacteria* (between 65 and 99% of the total sequences). In the whole data set, the most abundant OTU was affiliated with genus *Gallionella*. *Alpha- and Gamma-Proteobacteria* were the second and the third most abundant taxonomic groups.

The composition of the bacterial communities remained relatively stable in the AMD waters over the monitoring period with always a large dominance of reads related to *Gallionella* (ranging from 63 to 96%). Other minor groups identified in the waters included *Sulfuriferula, Acidithiobacillus* and *Ferrovum* (Supplementary Figure S2).

**FIGURE 4:**
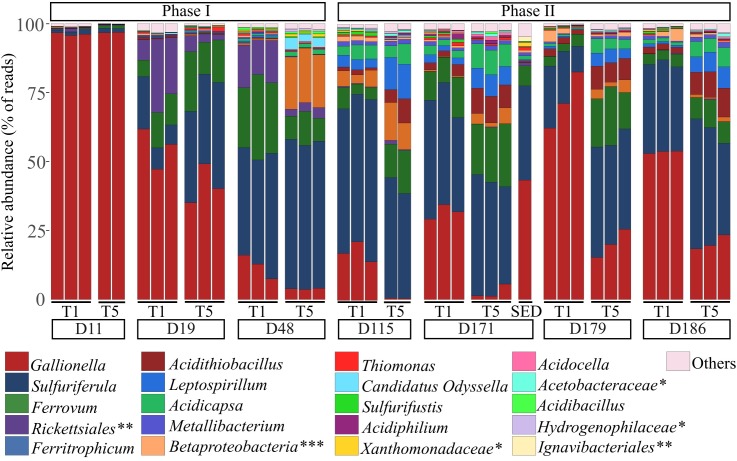
Taxonomic composition of bacterial communities (at the genus level) in the biogenic precipitates collected on the seven campaigns in the tray 1 (T1) and the tray 5 (T5) of the bioreactor (analyses performed in triplicates) and in the riverbed sediment (SED) collected on day 171. When genus identification was not possible, classification was made at the family level (^∗^), the order level (^∗∗^) or the class level (^∗∗∗^). “Others” represent the phylogenetic groups with a relative abundance < 1% calculated on the whole dataset.

Eleven days after the pilot was set up (D11), the bacterial communities of the biogenic precipitates were quite similar to those of the feeding water and largely dominated by the *Gallionella* genus (Figure 4). Then, notable temporal shifts rapidly occurred in the composition of the bacterial communities of the biogenic precipitates (phase I). From the 29th of June (D19), the proportion of *Gallionella* strongly decreased while those of *Sulfuriferula, Ferrovum, Rickettsiales, Ferritrophicum* and *Acidithiobacillus* increased. *Sulfuriferula* was dominant in the pilot between D48 and D171. From D115, the bacterial communities in the top tray and in the bottom tray were clearly distinct (phase II). The composition of the communities in T5 remained relatively stable, whereas in T1, the relative proportion of *Gallionella* increased again during this second phase of the monitoring (Figure 4).

Overall, for the whole monitoring period, the top tray was characterized by higher proportion of *Gallionella* compared to the bottom tray. *Sulfuriferula, Ferrovum, Ferritrophicum, Acidithiobacillus, Leptospirillum* and *Acidicapsa* were more abundant in the bottom tray. Spatial structuring inside the pilot was confirmed with the characterization of the bacterial communities in the five trays for two sampling campaigns (D48 and D171). This analysis showed that communities thriving in tray 2 were comparable to those in tray 1 whereas communities thriving in trays 3 and 4 were comparable to those in tray 5 (Supplementary Figure S3).

The composition of the bacterial community in the sediments (SED) collected in the AMD riverbed at D171, was similar to that of the biogenic precipitates collected the same day inside the top tray (T1) of the bioreactor (Figure 4). The relative proportion of *Thiomonas* in the Reigous creek sediments was comparable to that in the top tray of the treatment device (1.1% and 0.89 ± 0.25% respectively). The proportion of *Acidithiobacillus* was higher in the treatment pilot (2.8 and 9% respectively in tray 1 and 5) than in the riverbed sediment (0.6%).

### Quantification of Thiomonas Populations by CARD-FISH

Total bacteria and two distinct monophyletic groups of *Thiomonas*-related bacteria were quantified in the biogenic precipitates sampled in T1 and T5 at D48 (28th of July) and D171 (28th of November) by CARD-FISH (Table 1 and Supplementary Figure S4). Biogenic precipitates contained an average of 8 ± 3 10^7^ total bacterial cells/g of dry biogenic precipitates. No significant differences (*p* > 0.05) were observed for the total number of bacteria among the four samples analyzed. The total number of *Thiomonas* averaged 7 ± 2 10^6^ cells/g of dry biogenic precipitates, representing 8% of the DAPI-stained cells. *Thiomonas* belonging to group II (as defined by Bryan et al., 2009) were more abundant in the pilot than *Thiomonas* belonging to group I (*p* < 0.05). They corresponded respectively to an average of 4.9 and 3.1% of the DAPI-stained bacteria (Table 1). If no temporal differences were observed between D48 and D171, the total number of *Thiomonas* (Group I + II) was significantly higher in the bottom tray (T5) compared to the top tray (T1) in November (D171) (*p* < 0.05).

**Table 1:** Quantification by CARD-FISH of *Thiomonas* belonging to Group I (bacteria hybridized with the oligonucleotide probe TM2G0138) and of *Thiomonas* belonging to Group II (bacteria hybridized with the oligonucleotide probe TM1G0138).

	*Thiomonas* Group I		*Thiomonas* Group II		Bacteria (EUB I-III)	

Sample name	No of cells/g of dry biogenic precipitate	% of DAPI-strained cells	No of cells/g of dry biogenic precipitate	% of DAPI-strained cells	No of cells/g of dry biogenic precipitate	% of DAPI-strained cells
D48-T1	2 ± 1 × 10^6^	3 ± 2	5 ± 3 × 10^6^	6 ± 2	6.1 ± 0.7 × 10^7^	88 ± 1
D48-T5	3 ± 2 × 10^6^	4 ± 3	5 ± 3 × 10^6^	5 ± 3	7 ± 5 × 10^7^	82 ± 1
D171-T1	1.0 ± 0.3 × 10^6^	2.0 ± 0.7	3.4 ± 0.2 × 10^6^	4.6 ± 0.9	6 ± 1 × 10^7^	87 ± 2
D171-T5	4 ± 3 × 10^6^	4 ± 2	6 ± 2 × 10^6^	5 ± 2	1.2 ± 0.8 × 10^8^	83 ± 3


*Standard deviations were calculated on biological triplicates.*

### Main Drivers Shaping the Bacterial Communities Inside the Pilot

**FIGURE 5:**
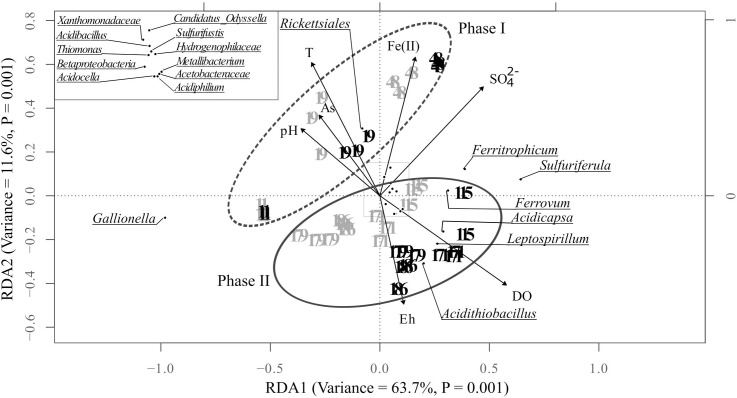
Redundancy analysis (RDA) illustrating the relationships between the environmental variables (DO, dissolved oxygen; SO_4_^2-^, dissolved sulfate concentration; T, temperature; pH, As, dissolved arsenic concentration; Fe(II), dissolved ferrous iron concentration and the redox potential Eh) and the distribution of the major bacterial taxa in the bioprecipitates collected on the seven campaigns in the tray 1 (gray) and the tray 5 (black) of the bioreactor (only the taxa with a relative abundance ≥ 1% in at least one bioprecipitate sample are included in this analysis). The analyses were performed in triplicates. The inset represents a close-up view of the center part of the RDA plot.

Redundancy analysis (RDA) summarized the spatio-temporal dynamics of the bioprecipitates bacterial communities and highlighted the main environmental factors explaining these variations. The samples were arranged according to the same pattern as in the NDMS (Figure 5). The model explained significantly 83.3% of bacterial community variation (Monte Carlo test, *P* < 0.05). According to variation partitioning, the dissolved oxygen (DO) was individually the best driver of this variability accounting for 36.5% of the total variance. It was followed by sulfate concentration (26.7%), temperature (16.5%), pH (15.2%), dissolved arsenic and ferrous iron concentration (11.1% and 10.2%, respectively) and the redox potential Eh (6%).

The bacterial communities thriving in the pilot during the first phase were clearly distributed along a temporal gradient and were associated with high values of temperature, pH, dissolved arsenic, and ferrous iron concentrations, and by low values of DO and Eh. Between D115 and D186, samples were divided into two sub-groups corresponding respectively to tray 1 and tray 5. During this second phase, the structure of the bacterial communities was explained by high DO and Eh values, particularly in the tray 5 (Figure 5).

The cosine of the angle between an environmental variable and a bacterial taxa provides an approximation of their correlation. Thus, it can be inferred from Figure 5 that the relative abundance of *Rickettsiales* was positively correlated with water temperature and negatively with Eh. On the contrary, *Acidithiobacillus, Leptospirillum* and *Acidicapsa* were negatively correlated with temperature, pH and arsenic concentration in water and positively with Eh and DO. For the other taxa, no clear correlations could be drawn.

## Discussion

In the *in situ* treatment device, iron and arsenic removal was driven by a microbial biofilm colonizing the surface of the five trays composing the pilot. The present study aimed at characterizing the bacterial diversity and its spatio-temporal dynamics in this engineered system, in relation with main environmental parameters.

### Microbiology of the Bioprecipitates

The biogenic precipitates that developed inside the pilot contained at least one order of magnitude higher bacterial biomass than what was observed in a lab-scale pilot treating the same AMD (Fernandez-Rojo et al., 2017) and in terraced iron formations reported elsewhere (Brown et al., 2011; Brantner et al., 2014). Thus, both the sand used to fill the trays and the hydrodynamic conditions were favorable to a biofilm development. The microbial assemblages developing inside this engineered system reproduced, at least partially, the natural ecosystem established in the nearby AMD. The microbiology of the pilot was largely dominated by diverse iron-oxidizing bacteria (FeOB): *Gallionella, Ferrovum, Ferritrophicum* and, to a lesser extent, *Acidithiobacillus* and *Leptospirillum.* The main taxa identified were previously found in AMD ecosystems (Baker and Banfield, 2003; Baker et al., 2003; Méndez-García et al., 2015; Chen et al., 2016), including Carnoulès (Bertin et al., 2011; Bruneel et al., 2011; Volant et al., 2014), and in laboratory-based experiments (Fernandez-Rojo et al., 2018; Tardy et al., 2018).

*Gallionella* proved to be dominant in the Carnoulès ecosystem (Bruneel et al., 2006; Volant et al., 2014; Tardy et al., 2018) and in a lab-scale pilot treating Carnoulès water (Fernandez-Rojo et al., 2018). Genus *Ferrovum*, containing litho-autotrophic acidophilic FeOB with potential biotechnological relevance (Heinzel et al., 2009a,b; Hedrich and Johnson, 2012), was also described in diverse AMD, including iron-rich environments (Johnson et al., 2014). *Gallionella* and *Ferrovum* were dominant in pilot plants relying on biological iron oxidation (Heinzel et al., 2009a,b; Hedrich et al., 2011; Hedrich and Johnson, 2012; Tischler et al., 2014; Sheng et al., 2016; Sun et al., 2016). *Ferritrophicum* genus, also including FeOB (Weiss et al., 2007), was previously identified in AMD (González-Toril et al., 2011; Tardy et al., 2018).

The moderately acidic sulfur oxidizers *Sulfuriferula* spp. were described as potential key actors of sulfur-cycling in acidic mine waste containing sulfide minerals (Jones et al., 2017). Their abundance in the pilot may be linked to the inflow of sulfide particles from the tailing pile into the pipe carrying the AMD water. The recent taxonomic reclassification of *Thiobacillus plumbophilus* to *Sulfuriferula plumbophilus* (Watanabe et al., 2015) may explain why *Sulfuriferula* spp. were underestimated so far in AMD. The capacity to oxidize iron or arsenic has not been described for any known *Sulfuriferula* species. “Sulfur-driven As(III) oxidation” was suggested in alkaline mono lake, through microbial thioarsenate transformation (Fisher et al., 2008). However, it is unlikely that similar process occurs under acidic and fully oxic conditions. Understanding the role of this genus (which mostly dominated the system from D48) in the treatment process requires further investigations.

*Acidithiobacillus* is considered as an important player in the attenuation process in Carnoulès (Egal et al., 2009; Bruneel et al., 2011; Bertin et al., 2011). This FeOB was detected in variable but relatively low proportions in the bioprecipitate (from 0.03 to 10.5%), in agreement with previous *in situ* observations (Volant et al., 2014). Sensibility to Fe(II) could explain the low abundance of *Acidithiobacillus* and the dominance of *Ferrovum* and *Gallionella* in the field-pilot where Fe(II) concentrations ranged between 649 and 1396 mg L^-1^ (Jones et al., 2015). The importance of *Acidithiobacillus* in biooxidation plants has previously been questioned (Rawlings et al., 1999). Even when used as an inoculum, *A. ferrooxidans* was out competed by *Ferrovum* in a pilot plant treating acid mine waters (Heinzel et al., 2009a). Distinguish the specific contribution of each FeOB population to iron and arsenic removal in the present study would require further investigations.

The presence of iron-reducing bacteria (*Acidiphilium, Acidocella, Metallibacterium*) can be explained by the occurrence of anoxic niches inside acidophilic biofilm (Ziegler et al., 2013).

### Dynamics of the Bacterial Communities Inside the Pilot

During the monitoring, biofilm colonization (phase I) was followed by a stationary phase (phase II) reached by the mature biofilm, and characterized by a stable bacterial biomass. The dominance of *Gallionella* in the bioprecipitates at the very beginning of the monitoring was linked to their dominance in the water used to feed the pilot. As the biofilm matured, spatial heterogeneities arose, resulting in the diversification of micro-habitats advantageous for other populations (Santegoeds et al., 1998; Woodcock and Sloan, 2017). The proportion of *Sulfuriferula, Ferrovum, Ferritrophicum, Rickettsiales* increased while those of the first colonizers *Gallionella* decreased. Increase of bacterial diversity and shifts in bacterial assemblages were previously described during acidophilic biofilm maturation (Wilmes et al., 2009; Mueller et al., 2011).

*Ferrovum myxofaciens* produces large amounts of extracellular polymeric substances, which facilitate attachment to surfaces (Rowe and Johnson, 2008). This capacity may provide a competitive advantage to *Ferrovum*-related bacteria in engineered systems. In two pilot plants treating acid mine water, *Gallionella* were dominant during a first phase associated with unstable operating conditions while *Ferrovum* became dominant during a second phase with stable conditions. *Gallionella* abundance increased again at the end of the monitoring (Heinzel et al., 2009b). Similarly, in the present study, *Gallionella* regained in importance during the later stages of the monitoring, particularly in the top tray.

The autochthonous bacterial communities of the AMD feeding the pilot remained stable and largely dominated by *Gallionella* during the 6 months monitoring. Thus, the temporal dynamic of the bacterial community in the bioprecipitate (particularly during phase I) can be more readily attributed to bacterial succession during biofilm establishment rather than to the modification of the seeding community.

During the second phase of the monitoring, the spatial structuring of the mature communities inside the pilot intensified. Differences observed between the top and the bottom trays can be linked to a modification of the chemistry of the overlying water while transiting inside the pilot. This chemical gradient between the inlet and the outlet water was particularly marked during this second phase (Supplementary Table S1).

### Influence of Environmental Parameters on the Bacterial Communities

The range of physico-chemical variations observed in the AMD water during the 6 months monitoring was representative of the seasonal variations described during a 4 years *in situ* monitoring (Egal et al., 2010). The present study covered both summer (phase I) and autumn (phase II) periods. Thus, a seasonal effect on the whole dynamic may not be excluded. However, both autogenic (e.g., internal and biotic) and allogenic (e.g., external and abiotic) parameters are likely to govern bacterial dynamics during biofilm establishment. It is then difficult to distinguish the effect of seasonal variations from the autogenic parameters that may be particularly influential during the early phases of the biofilm development (Jackson et al., 2001; Lyautey et al., 2005). However, we can assume that once the biofilm has reached its maturity, the bacterial community dynamic was mainly driven by the variations of the physico-chemistry of the overlying water (Brown et al., 2011).

Redundancy analysis highlighted the key parameters susceptible to influence bacterial diversity in the pilot: DO, sulfate concentration, temperature, pH, dissolved As and Fe(II) concentration, and Eh (Rawlings et al., 1999; Dopson et al., 2007; Kupka et al., 2007; Candy et al., 2009; Volant et al., 2014; Jones et al., 2015; Sheng et al., 2016).

*Gallionella* is a microaerophilic bacteria thriving at lower oxygen conditions than other FeOB as *Ferrovum, A. ferrooxidans* and *Leptospirillum* spp. (Hanert, 2006; Johnson et al., 2014). The persistence of microaerophilic bacteria in the bioprecipitates and in the sediments, including some *Gallionella* and *Ferritrophicum* (Weiss et al., 2007), could be explained by the possible occurrence of microaerobic niches, as seen elsewhere (Ziegler et al., 2013). On the contrary, higher abundances of *Ferrovum* were associated with increasing concentration of oxygen (Jwair et al., 2016; Fabisch et al., 2016; Fernandez-Rojo et al., 2018).

Most of the FeOB characterized so far are mesophilic or thermophilic (Kupka et al., 2007). A better tolerance to low temperatures of *Acidithiobacillus* (Norris, 1990) may explain why its abundance was negatively correlated with temperature in the pilot. Batch experiments conducted with Carnoulès AMD water showed a clear effect of temperature on arsenite-oxidizers: both the abundance of *Thiomonas* and the As(III) oxidation activity were stimulated at 35°C compared to 20°C (Tardy et al., 2018). In the temperature range covered by the present study (5.2-23.8°C), no correlation was evidenced between temperature and *Gallionella, Ferrovum* or *Thiomonas* relative abundances. Temperatures naturally reaching 30°C or more in the water column above the bioprecipitate during summer are not excluded, particularly in case of reduced water height and long residence times. Such high temperatures may have differential effect on the FeOB and AsOB activity depending on their physiology.

Regarding the pH, our results are congruent with the better adaptability of *Ferrovum, Acidithiobacillus*, and *Leptospirillum* to acidic conditions compared to *Gallionella* known to prefer pH above 3 (Rawlings et al., 1999; Jones et al., 2015; Grettenberger et al., 2017).

Fe(II) concentration also influences growth and activity of FeOB depending on their affinity for this energy source and on potential competition between different populations (Battaglia et al., 1994; Rawlings et al., 1999). The negative correlation of *Acidithiobacillus* with Fe(II) is in agreement with a previous study (Jones et al., 2015). Furthermore, different affinity for Fe(II) together with distinct sensitivity to Fe(III) inhibition may explain the effect of redox potential (conditioned by the ratio Fe(III)/Fe(II)) on the relative distribution of FeOB (Rawlings et al., 1999; Meruane et al., 2002; Kuang et al., 2013).

Occurrence of *Gallionella* was negatively correlated with sulfate concentrations. Sulfates are generally well tolerated by FeOB, with inhibitory effects reported for concentrations higher than the ones measured in the present study (Candy et al., 2009). However, sulfate concentrations were previously shown to drive bacterial community composition in the Carnoulès AMD waters (Volant et al., 2014).

Finally, dissolved arsenic concentration significantly contributed to the bacterial community structure inside the bioreactor. Similar influence was shown *in situ* (Volant et al., 2014). Nevertheless, no tendency was highlighted between the variations of dissolved arsenic concentration and the proportion of the arsenite-oxidizing *Thiomonas*. It can be hypothesized that in the range of concentrations measured in this study, arsenic was not a limiting factor for bacteria relying on arsenite oxidation for their growth, and was not toxic for the others *Thiomonas*. *Acidithiobacillus* appeared to be negatively correlated with dissolved arsenic concentration although high resistance capacity was evidenced for some strains of *A. ferrooxidans* (up to 15 g L^-1^ As(III), Dave et al., 2008). A possible explanation is that *Acidithiobacillus* contribute to arsenic removal from water and its abundance in the bioprecipitates is thus associated with lower dissolved arsenic concentrations.

### Iron Oxidation and Precipitation

In spite of the large dominance of FeOB in the pilot, iron oxidation and precipitation did not exceed 20% and 11%, respectively (Fernandez-Rojo et al., in press). Iron-oxidizing activity of *A. ferrooxidans* was strongly inhibited when the bacteria were attached to solid surfaces (Wakao et al., 1984). On the contrary, *Ferrovum* biofilms proved to be very effective in iron-oxidizing bioreactors (Hedrich and Johnson, 2012). Among several strains tested by Johnson et al. (2012) the highest iron oxidation rates were obtained with *Ferrovum* spp. In spite of their well-known efficiency and their adaptation to temperature and pH ranges observed in the pilot (Kuang et al., 2013; Johnson et al., 2014; Jones et al., 2015), the iron-oxidizing activity of FeOB (including *Ferrovum*) appeared limited in the present study possibly because of non-optimal operating conditions. The water height above the biofilm (1.5–7 cm), which is a key factor for iron oxidation kinetics, may have prevented a sufficient oxygenation of the water column (Brown et al., 2011; Fernandez-Rojo et al., 2017). Another possible limiting factor is an insufficient active surface of the biofilm in regard to the volume of overlying water to be treated. Probably due to these limitations, the important variations of FeOB distribution inside the pilot did not result in significant iron oxidation rate variation (Figure 1B). Our results strongly suggest that the composition of the bacterial communities was not a limiting factor for iron oxidation in the bioreactor. Further work based on RNA will help to determine which bacterial taxa actively contribute to the treatment.

### Bacterial Arsenite Oxidation in the Pilot

As(III) oxidation by *Thiomonas* spp. can contribute to the formation of stable As(V)-rich precipitates. No clear temporal or spatial tendency was observed for *Thiomonas* genus based on metabarcoding. Further insights into the abundance and the identity of the *Thiomonas* spp. thriving in the pilot were gained by a CARD-FISH approach. In the biogenic precipitates sampled at D48 and D171, the proportion of *Thiomonas* related bacteria averaged 8% of the DAPI-stained bacteria, which is congruent with *in situ* observations (5-8.7%, Hovasse et al., 2016). Despite their low abundance, *Thiomonas*-related bacteria are considered to play a crucial role in the pollution mitigation (Bruneel et al., 2003, 2011; Hovasse et al., 2016). Metaproteomic approaches showed that they sustainably express an arsenite oxidase activity *in situ* in Carnoulès (Bertin et al., 2011; Hovasse et al., 2016). Inside the pilot, the *Thiomonas* belonging to group II (as defined by Bryan et al., 2009) were more abundant than those belonging to group I (*p* < 0.05), whereas the average proportions of the two groups were similar *in situ* (Hovasse et al., 2016). Both groups I and II contain arsenite-oxidizing *Thiomonas* strains isolated from the Carnoulès AMD (Bryan et al., 2009; Bertin et al., 2011; Hovasse et al., 2016). Group II includes *Thiomonas* sp. CARN2, which expressed its arsenite oxidation activity in the Carnoulès AMD sediments (Bertin et al., 2011). The As(III)-oxidation genetic potential (expressed as the number of *aioA* genes ng^-1^ of DNA) increased tenfold in the bioprecipitate during the first 48 days suggesting the establishment of an active As(III)-oxidizing bacterial population during the early stages of the biofilm maturation (Fernandez-Rojo et al., in press). Although the detection of *aioA* genes doesn’t give evidence of its expression, we can hypothesize that the *Thiomonas*-related bacteria identified by CARD-FISH were responsible for the arsenic oxidation resulting in the formation of solid phases containing almost 100% of As(V).

## Conclusion

The present study gave new insights into the bioremediation, at the field-scale, of As-rich AMD by biological iron and arsenic oxidation. Bacterial communities originating from the AMD water and organized in biofilm inside the pilot successfully removed soluble arsenic. It can be assumed that, during the first phase of the monitoring, the temporal dynamic of the biogenic precipitates communities was mostly due to ecological succession during biofilm installation. Once the biofilm was mature, the physico-chemistry of the overlying water exerted a complex control on the distribution of the biofilm bacterial populations. The co-existence of several FeOB populations characterized by distinct physiological traits (in term of optimal pH, temperature, DO, and Fe(II) affinity…) permitted a good adaptation of the system toward variations in the chemistry of the AMD water to be treated. Ubiquity of the bacteria identified and the presence of bacteria of biotechnological relevance (*Ferrovum, Acidithiobacillus*) let expect that the application of this system to other As-and Fe-rich AMD worldwide is practicable.

We showed evidence of the stable presence of distinct populations of *Thiomonas* spp. in the pilot. Appreciable arsenite oxidation occurred in the field pilot. To determine the factors controlling arsenite oxidation activity in this treatment system, gene expression investigations are required.

The development of accurate biological treatment requires the stability of bacterial activity under seasonal variations. Furthermore, the bacterial diversity of the AMD water remained relatively stable during the 6 months monitoring whereas it proved to be more variable over a longer period of time (Volant et al., 2014). For these reasons, a monitoring on a longer period is required to guarantee the long-term stability and robustness of the treatment system.

## Author Contributions

CC and MH supervised the research project and the experiments. EL performed the metabarcoding analyses and interpretations. AD performed the CARD-FISH. CJ, FB-B, OB, and VT contributed to data interpretation. CC, EL, LF-R, MH, OB, and VT collected the samples. MH, EL, and CC wrote the paper. All authors read and approved the final manuscript.

## Conflict of Interest Statement

The authors declare that the research was conducted in the absence of any commercial or financial relationships that could be construed as a potential conflict of interest.

## Footnotes

1 http://www.r-project.org/
