# Supplementary material for: Dynamics of Bacterial Communities Mediating the Treatment of an As-Rich Acid Mine Drainage in a Field Pilot
Source: Front Microbiol. 2018 Dec 21;9:3169. doi: 10.3389/fmicb.2018.03169 (PMC6309452; doi:10.3389/fmicb.2018.03169)
Supplement: Supplementary file 1 [file Data_Sheet_1.docx]

Supplementary Material

Dynamics of Bacterial Communities Mediating the Treatment of an As-Rich Acid Mine Drainage in a Field Pilot

**Laroche E., Casiot C., Fernandez-Rojo L., Desoeuvre A., Tardy V., Bruneel O., Battaglia-Brunet F., Joulian C., Héry M.***

***Correspondence:** [marina.hery@umontpellier.fr](mailto:marina.hery@umontpellier.fr)

# Supplementary data

## CARD – FISH

Biogenic precipitates were fixed in freshly prepared 4% paraformaldehyde [1:10 w/v] for 2 h 30 at 4°C, washed with phosphate buffered saline (PBS), and stored at -20°C in 50% PBS/ethanol solution.

### Oligonucleotide probes for hybridization

Probes (Biomers, Ulm, Germany) were labeled by horseradish peroxidase (HRP). The mixture of 16S rRNA targeted oligonucleotide probes EUB338 (5’GCTGCCTCCCGTAGGAGT-3’), EUB338II (5′-GCAGCCACCCGTAGGTGT-3′) and EUB338III (5′-GCTGCCACCCGTAGGTGT-3′) were applied for Bacteria detection (Amann 1990, Daims et al., 1999). Negative controls were performed with probe NON338 (5’-ACTCCTACGGGAGGCAGC-3’), the complementary sequence of EUB338 (Wallner et al., 1993). Very few (< 1%) or no probe-positive cells were detected using the negative control. Two probes were used for the quantification of two distinct monophyletic groups of *Thiomonas* spp. Probes TM1G0138 (5′-GCAGTTATCCCCCATCAAT-3’) and TM2G0138 (5’- GTAGTTATCCCCCATCACA-3’) (Hallberg et al., 2006) target respectively the monophyletic Group II and I according to Coupland et al. (2004) and Bryan et al. (2009).

### In situ hybridization

The first steps of the CARD-FISH procedure were conducted as described in Eickhorst and Tippkötter (2008), with some modifications. One hundred microliters of fixed sample were diluted in 900 µL MilliQ water (MQ), sonicated 35 s at 42 kHz, diluted in 9 mL of MQ and filtered on polycarbonate filters (0.2 µm, 47 mm in diameter, Merck Millipore, Darmstadt, Germany). The filter was dipped in 0.2% low melting point agarose and cells were permeabilized at 37°C during 1 h in lysozyme (10 mg mL^-1^) solution and then during 30 min in achromopeptidase solution (60 U mL^−1^). Endogenous peroxidase activity was inactivated by incubation in methanol containing 0.15% H_2_O_2_ for 30 min. Filter was dehydrated in 98% EtOH and air dried. Sections of filters were cut out and placed in 300 µL of hybridization buffer (0.9 M NaCl, 20 mM Tris–HCl [pH 8.0], 10% (w/v) dextran sulphate, 2% (w/v) blocking reagent (Roche, Mannheim, Germany), 0.1% (w/v) sodium dodecyl sulphate (SDS), 35% (v/v) formamide) containing 100 ng of HRP-labeled probes. After hybridization at 35°C for 2 h, a washing of not bound probes was performed: the slides were transferred into prewarmed washing buffer (3 mM NaCl, 5 mM EDTA [pH 8.0], 20 mM Tris–HCl [pH 8.0], 0.01% (w/v) SDS) at 37°C for 30 min followed by an incubation 15 min into 0.05% (v/v) Triton X100 amended PBS (PBS-T).

### Tyramide amplification

Tyramide amplification was modified from Schmidt et al. (2012). Tyramide and amplification buffer were home-made. For the tyramide solution, triethylamine (10 μL mL^−1^; Sigma-Aldrich) and dimethylformamide (DMF) were mixed with tyramine HCl (10 mg mL^−1^; Sigma-Aldrich). 6-(fluorescein-5-carboxamido) hexanoic acid, succinimidyl ester (SFX; Invitrogen) was dissolved in DMF (10 mg mL^−1^). The two solutions were mixed in the ratio of 1:3 (v/v) incubated for 6 h in the dark and diluted to a final concentration of 1 mg SFX mL^-1^ in DMF containing 2% [w/v] 3-iodophenolboronic acid (IPBA, Sigma-Aldrich) to a final concentration of 1 mg mL^-1^ (SFX-DMF).

For tyramide signal amplification, the filters sections were incubated for 15 min at 46°C in 500 μL amplification buffer AB2 (0.1 mg μL dextran sulfate, 1% blocking reagent, 2 M NaCl, 1×PBS) containing 0.0015% H_2_O_2_ and 0.5 µL of fluorescein-labeled tyramide home-made solution. Sections were then washed with PBS-T for 15 min in dark, rinsed with MQ three times and air-dried.

Sections with CARD-FISH-stained samples were mounted with Vectashield H-1200 (Vector Laboratories, Burlingame, California) containing 4’, 6-diamino-2-phenylindole (DAPI) to protect the fluorescence and to counterstain. The samples were analyzed using a microscope Axioimager Z2 apotome (Carl Zeiss, Jena, Germany). A minimum of 1,000 DAPI-stained cells per triplicate and per probe were counted on 15-25 randomly selected micrographs taken with objective 63x (44648 µm^2^) and extrapolated to 1 g of biogenic precipitated (dry weight). Detection rates were calculated by the fraction of DAPI-stained cells detected with CARD-FISH.

# Supplementary figures and tables

## Supplementary tables

| Sample name | pH | T  (°C) | Eh  (mV) | DO  (mg L^-1^) | EC  (mS cm^-1^) | TDS  (mg L^-1^) | Flow rate  (L h^-1^) | SO_4_^2-^  (mg L^-1^) | Fe(II)  (mg L^-1^) | Fe  (mg L^-1^) | As(III)  (mg L^-1^) | As(V)  (mg L^-1^) | As  (mg L^-1^) |
| --- | --- | --- | --- | --- | --- | --- | --- | --- | --- | --- | --- | --- | --- |
| D11-Win | 3.28 | 21.7 | 530 | 2.23 | 3.79 | 1968 | 31.50 | 784 | 957 | 978 | 92 | 22 | 113 |
| D11-Wout | 2.87 | 19.8 | 580 | 3.68 | 3.79 | 1961 | 39.12 | 701 | 896 | 890 | 56 | 11 | 67 |
| D19-Win | 3.36 | 18.6 | 522 | 3.18 | 3.78 | 1962 | 77.94 | 825 | 1010 | 966 | 91 | 19 | 118 |
| D19-Wout | 3.1 | 19.3 | 535 | 4.08 | 3.76 | 1860 | 81.35 | 932 | 959 | 957 | 72 | 16 | 98 |
| D48-Win | 3.21 | 19.5 | 527 | 2.8 | 3.91 | 2230 | 107.14 | 1078 | 1295 | 1187 | 107 | 23 | 152 |
| D48-Wout | 3.04 | 18.1 | 546 | 6.96 | 3.87 | 2200 | 125.47 | 1132 | 1218 | 1227 | 100 | 23 | 147 |
| D115-Win | 3.23 | 18.8 | 467 | 5.77 | 4.39 | 2300 | 0.28 | 1167 | 1396 | 1314 | 94 | 26 | 134 |
| D115-Wout | 2.82 | 17.9 | 590 | 6.66 | 4.55 | 2370 | 6.36 | 1237 | 1350 | 1262 | 0 | 3 | 3.8 |
| D171-Win | 2.64 | 12.5 | 563 | 5.22 | 4.33 | 2310 | 47.37 | 859 | 806 | 833 | 53 | 15 | 84 |
| D171-Wout | 2.67 | 5.2 | 599 | 8.84 | 4.06 | 2100 | 48.65 | 758 | 763 | 761 | 42 | 15 | 63 |
| D179-Win | 3.11 | 11.3 | 584 | 5.16 | 3.86 | 2060 | 41.66 | 815 | 693 | 789 | 62 | 15 | 86 |
| D179-Wout | 2.91 | 5.6 | 603 | 8.37 | 4.06 | 2110 | 35.10 | 827 | 663 | 751 | 35 | 11 | 51 |
| D186-Win | 3.41 | 10.1 | 573 | 5.49 | 4.32 | 2250 | 49.01 | 830 | 874 | 814 | 68 | 15 | 93 |
| D186-Wout | 3.11 | 5.4 | 598 | 9.58 | 3.95 | 2050 | 35.42 | 811 | 649 | 768 | 48 | 12 | 62 |

**Supplementary Table S1.** Physico-chemical characteristics of inlet (W_in_) and outlet (W_out_) waters tested as environmental variables in RDA analysis (T=temperature, Eh = redox potential, DO= dissolved oxygen concentration, EC = electrical conductivity, TDS = total dissolved solid, SO_4_^2-^= dissolved sulfate concentration, Fe(II)=dissolved ferrous iron concentration, Fe = dissolved iron concentration, As(III) = dissolved arsenite concentration, As(V) = dissolved arsenate concentration, As=dissolved arsenic concentration) (Fernandez-Rojo et al., 2019).

## Supplementary figures

**
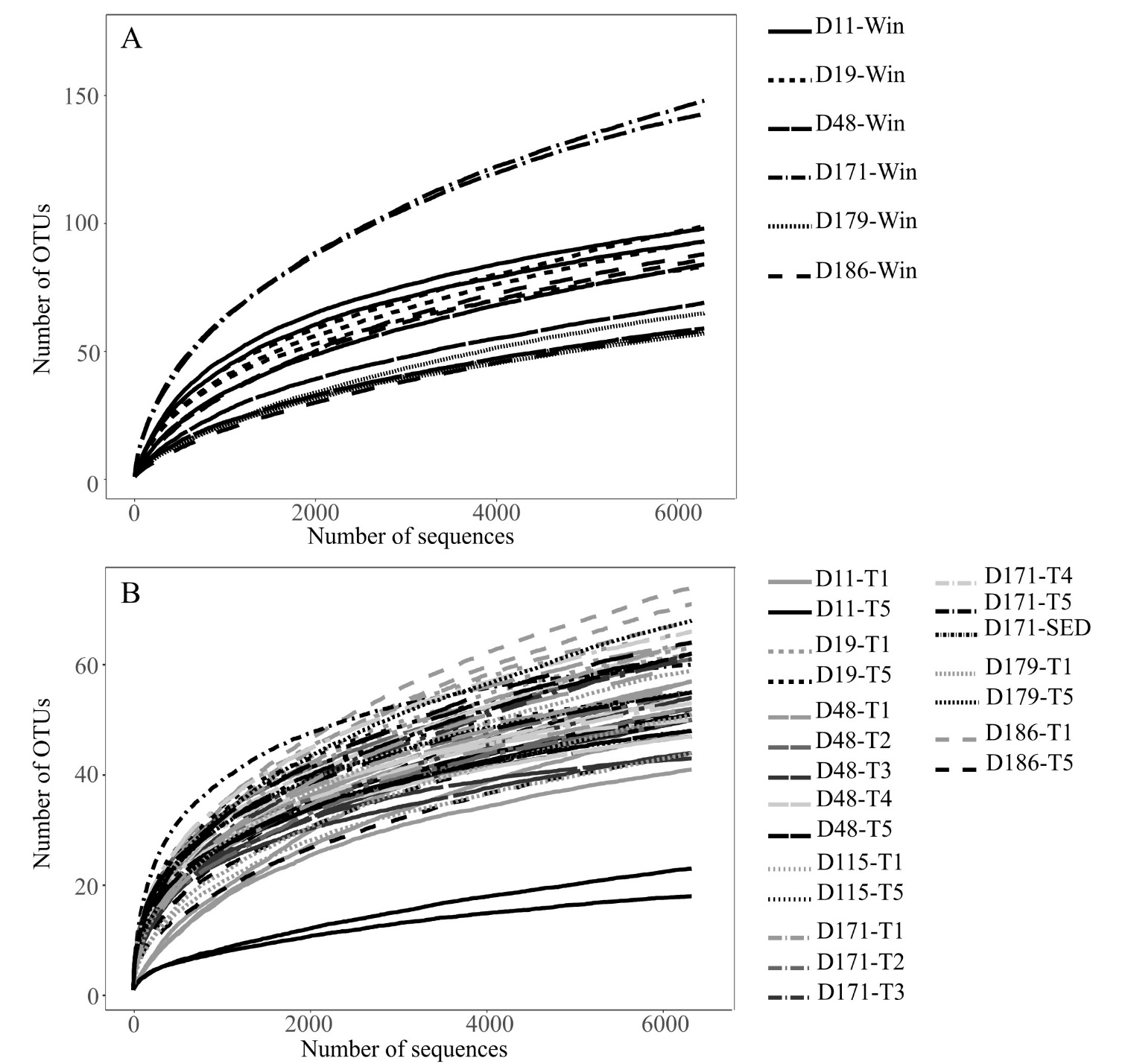
Supplementary figure S1.** Rarefaction curves comparing the number of detected OTUs with the number of bacterial 16S rRNA gene sequences from the inlet waters (A) and the biogenic precipitates collected in the trays of the bioreactor (B) during the whole monitoring period. Analyses were performed in triplicates.

**
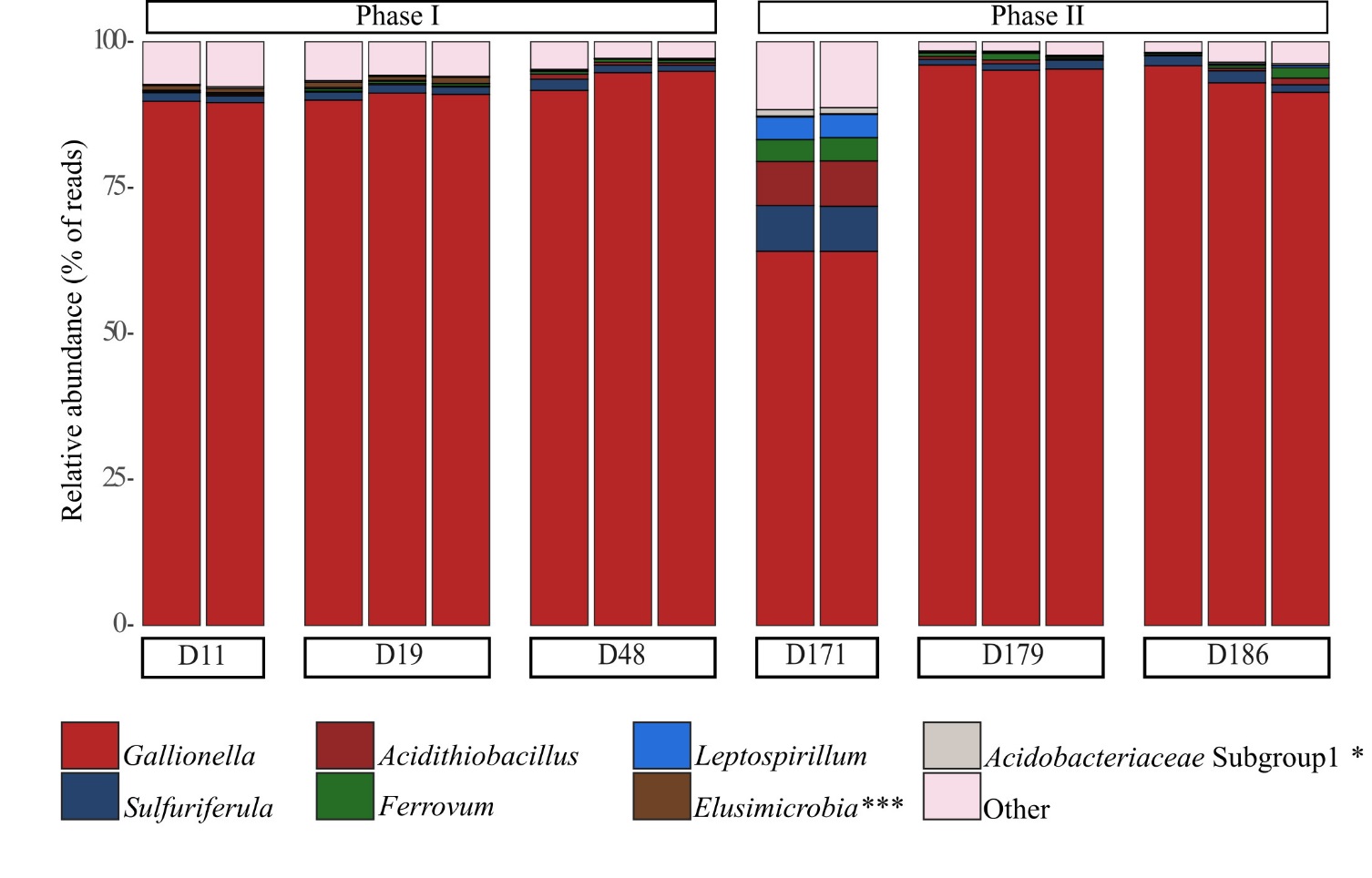
Supplementary figure S2.** Taxonomic composition of bacterial communities (at the genus level) in the inlet waters during the whole monitoring period (except D115). When genus identification was not possible, classification was made at the family level (*) or the class level (***). “Others” represent the phylogenetic groups with a relative abundance < 1% calculated on the whole dataset. Analyses were performed in triplicates.

**
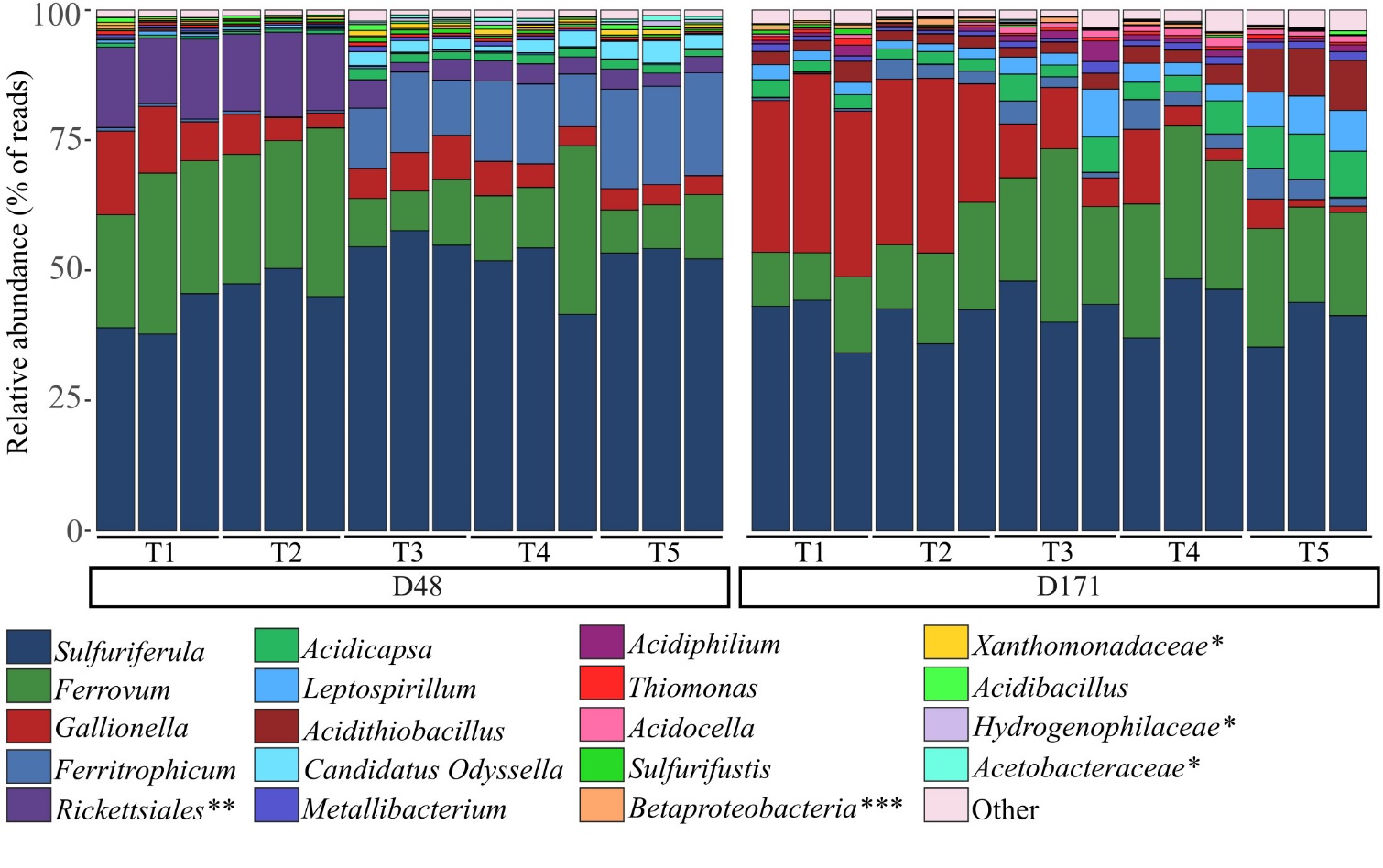
Supplementary figure S3**: Taxonomic composition of bacterial communities (at the genus level) in the biogenic precipitates collected on day 48 and on day 171 in all the trays (T1-T5). Analyses were performed on triplicates. When genus identification was not possible, classification was made at the family level (*), the order level (**) or the class level (***). “Others” represent the phylogenetic groups with a relative abundance < 1% calculated on the whole dataset.


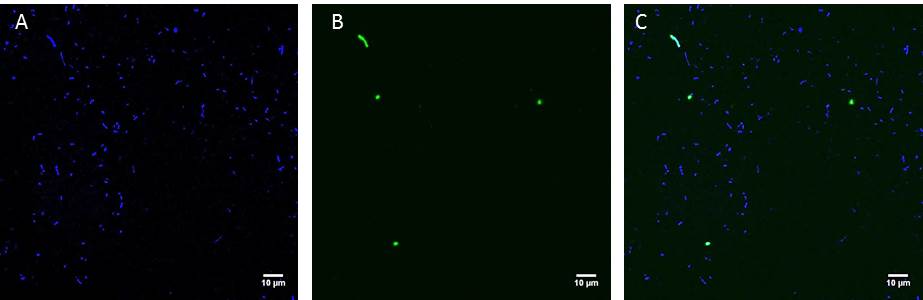


**Supplementary figure S4**: CARD-FISH photomicrographs of bacterial cells from biogenic precipitates collected at D171 (November 2016). DAPI-stained cells, in blue (A). CARD-FISH staining of *Thiomonas* Group II cells with TM1G0138 probe, in green (B). Merged images (C). Scale bar = 10 µm.

Reference

Amann, R.I., Binder, B.J., Olson, R.J., Chisholm, S.W., Devereux, R., and Stahl, D.A. (1990). Combination of 16S rRNA-targeted oligonucleotide probes with flow cytometry for analyzing mixed microbial populations. Appl. Environ. Microbiol. *56*, 1919–1925.

Bryan, C.G., Marchal, M., Battaglia-Brunet, F., Kugler, V., Lemaitre-Guillier, C., Lièvremont, D., Bertin, P.N., and Arsène-Ploetze, F. (2009). Carbon and arsenic metabolism in Thiomonas strains: differences revealed diverse adaptation processes. BMC Microbiol. *9*, 127. [doi:10.1186/1471-2180-9-127](https://doi.org/10.1186/1471-2180-9-127).

Coupland K., Battaglia-Brunet F., Hallberg K.B., Dictor M.-C., Garrido F. and Johnson D.B. (2004). Oxidation of iron, sulfur and arsenic in mine waters and mine wastes: an important role for novel Thiomonas spp. In: Tsezos M, Hatzikioseyian A & Remoudaki E. (eds). Biohydrometallurgy; a sustainable technology in evolution. National Technical University of Athens, Zografou, Greece, pp. 639–646

Daims, H., Brühl, A., Amann, R., Schleifer, K.H., and Wagner, M. (1999). The domain-specific probe EUB338 is insufficient for the detection of all Bacteria: development and evaluation of a more comprehensive probe set. Syst. Appl. Microbiol. *22*, 434–444. [doi:10.1016/S0723-2020(99)80053-8](https://doi.org/10.1016/S0723-2020(99)80053-8).

Eickhorst, T., and Tippkötter, R. (2008). Improved detection of soil microorganisms using fluorescence in situ hybridization (FISH) and catalyzed reporter deposition (CARD-FISH). Soil Biology and Biochemistry 40 (7): 1883 91. <https://doi.org/10.1016/j.soilbio.2008.03.024>.

Hallberg, K.B., Coupland, K., Kimura, S., and Johnson, D.B. (2006). Macroscopic streamer growths in acidic, metal-rich mine waters in north wales consist of novel and remarkably simple bacterial communities. Appl. Environ. Microbiol. *72*, 2022–2030. [doi:10.1128/AEM.72.3.2022-2030.2006](https://doi.org/10.1128/AEM.72.3.2022-2030.2006).

Schmidt, H., Eickhorst, T. and Tippkötter, R. (2012). Evaluation of Tyramide Solutions for an Improved Detection and Enumeration of Single Microbial Cells in Soil by CARD-FISH. *Journal of Microbiological Methods* 91 (3): 399‑405. <https://doi.org/10.1016/j.mimet.2012.09.021>.

Wallner, G., Amann, R., and Beisker, W. (1993). Optimizing fluorescent in situ hybridization with rRNA-targeted oligonucleotide probes for flow cytometric identification of microorganisms. Cytometry *14*, 136–143. [doi:10.1002/cyto.990140205](https://doi.org/10.1002/cyto.990140205).
